# Ammonia fumigation combined with nitrogen inhibitor reduces root-knot nematode populations and N_2_O emission but not bacterial alpha diversity

**DOI:** 10.3389/fmicb.2026.1756683

**Published:** 2026-07-22

**Authors:** Fengyu Shi, Jiawei Fu, Pengyuan Li, Xiaotong Li, Jianbin Liu, Yingbo Zhu

**Affiliations:** 1 Hebei Key Laboratory of Crop Stress Biology, Hebei Normal University of Science and Technology, Qinghuangdao, China; 2 Institute of Plant Nutrition, Resources and Environment, Beijing Academy of Agriculture and Forestry Sciences, Beijing, China

**Keywords:** ammonia fumigation, inhibitors, nitrogen oxides, root-knot nematodes, soil microbial community

## Abstract

Aqueous ammonia can release high nitrogen inorganic substances of ammonia and has been used as soil fumigant. However, the information about how fumigation with ammonia affects the soil microbial community is still limited. In this study, a laboratory microcosm experiment was conducted to evaluated the effectiveness of ammonia fumigation in controlling root-knot nematodes (RKNs), its influence on soil microbial diversity, and its impact on nitrous oxide (N_2_O) emissions. The results indicated that Ammonia treatment increased N_2_O emissions and decreased the RKNs population by 75.73%. Adding the inhibitors N-(N-butyl) thiophosphoric triamide (NBPT) and Dicyandiamide (DCD) to aqueous ammonia can mitigate N_2_O emissions. Compared with CK treatment, Soil enzyme activities, including those of urease, nitrate reductase, and nitrite reductase, were enhanced in each treatment. The fungal Sobs index was significantly higher in CK treatment than in the other treatments, whereas bacterial alpha diversity remained unaffected. This study also indicates that soil fumigation with aqueous ammonia has the potential to regulate fungal communities and important key reactions involved in nitrogen cycling.

## 1 Introduction

Root-knot nematodes (*Meloidogyne* spp.) are the significant plant parasites responsible for annual global agricultural losses of approximately $118 billion, infecting nearly all crops (Atkinson et al., 2012; Elling, 2013; Yadav et al., 2020; Meza Zamora et al., 2024). More than 40 species of root-knot nematodes (RKNs) have so far been reported across more than 27 provinces in China (Shen et al., 2023). Among the RKNs, *Meloidogyne incognita* and *M.hapla* are considered the most important phytopathogens infecting vegetable crops. In severe infestations, galled roots of vegetable crops may rot away resulting in death. It has become a serious threat to the production of vegetable crops.

At present, the control methods to RKNs are mainly chemical, physical, and biological disinfection (Azeem et al., 2020; Ahmed, 2021; Haq et al., 2022). Physical methods, such as high-temperature soil steaming, flame, microwave and ultraviolet disinfection, are typically applied during the summer to target surface soil layers (Chang et al., 2023). However, these methods often fail to effectively eliminate pathogens from deeper soil layers. Although hot water injection is effective, its labor-intensive nature and high cost limit its feasibility for large-scale application (Laquale et al., 2015). The biological method, such as biofumigation technology, relies on the volatile compounds released during the decomposition of plants or organic materials, which are primarily used by *Brassicaceae* and *Asteraceae* plants to inhibit or kill soil pathogens (D’Addabbo et al., 2014; Baglioni et al., 2024). However, its effectiveness in controlling RKNs is heavily influenced by environmental conditions and typically requires a longer duration to achieve noticeable results (Omirou et al., 2011; Castellano-Hinojosa et al., 2022).

Chemical soil fumigation remains one of the most effective and widely used method for controlling soil-borne diseases and pests, including RKNs. However, fumigation can affect both pathogenic and non-target microorganisms in the soil. As microbial communities are critical within the soil ecosystem, depletion of or changes in these communities can result in negative implications for soil health. Previous studies have shown that certain fumigants, including 1,3-dichloropropene (1,3-D), chloropicrin and methyl bromide have varying effects on soil microbial communities (Stromberger et al., 2005; Yan et al., 2015; Jiang et al., 2022). Recent studies showed that chloropicrin and metham sodium caused a significant change in the population size and composition of bacteria involved in nitrogen cycling (Li et al., 2017a,b). In addition, many studies reported that chloropicrin increased total N_2_O emissions by 23–25 times by inhibiting the expression of gene families involved in N_2_O reduction, thereby shifting the main pathway of N_2_O production from nitrification to denitrification (Spokas et al., 2005, 2006; Yan et al., 2015; Fang et al., 2018a,b).

As an essential inorganic nutrient for crops, ammonia has demonstrated effectiveness as a cost-efficient fumigation compared to chemical fumigants. Ammonia effectively controls soil-borne pathogens, such as *Fusarium oxysporum, Verticillium dahliae*, *Gaeumannomyces graminis*, and numerous weed seeds in the soil (Bashour et al., 2013). Aqueous ammonia has been shown to eliminate *F.solani* spores, the pathogen responsible for wheat crown rot, and reduce *Fusarium* wilt in banana soils (Schippers and Palm, 1973). Additionally, Aqueous ammonia has been reported to control *M.incognita* (Su et al., 2015). However, as a volatile nitrogen fertilizer, it is unclear how aqueous ammonia fumigation influences root-knot nematodes, the population abundance and community structure of soil microorganisms and N_2_O emissions. Thus, the aims of this study were to (1) assess the effects of ammonia fumigation on the population of RNKs and N_2_O production; (2) examine the effect of nitrogen inhibitor added to ammonia-fumigated soils on N_2_O emission; (3) investigate the effects of ammonia fumigation on soil microbial diversity using High-throughput sequencing technology.

## 2 Materials and methods

### 2.1 Soil sampling collection

Soil (0–20 cm depth) was collected in mid-May 2024 during cucumber harvest at Gaocheng District, City, Hebei Province, China (38°1′24.9″N, 114°50′26.2″E). The RKN-infested field had no previous history of fumigation treatment. Soil samples were sieved through a 2 mm sieve and stored at 4 °C before any treatment. The soil’s physicochemical properties were as follows: the electrical conductivity (EC, soil-to-water ratio of 1:5, w/v) of 0.35 ± 0.01 mS cm^−1^, pH (soil-to-water ratio of 1:2.5, w/v) of 7.0 ± 0.04, the total nitrogen of 2.29 ± 0.12 g kg^−1^, NO_3_^−^-N of 17.29 ± 0.82 mg kg^−1^, NH_4_^+^-N of 6.5 ± 0.51 mg kg^−1^. The initial population of root-knot nematodes was 910.67 ± 70.2 individuals per 100 g of dry soil.

### 2.2 Experimental design

The microcosm experiment was set up in a 500 mL wide-neck glass bottle (Beijing Zhongke Sicheng Technology Co., Ltd.). The soil (350 g) was adjusted to 40% water filled pore space (WFPS), and then pre-incubated for 7 d at room temperature in the dark before transfer to each microcosm. The height of soil in the glass bottle was 3.8 cm. Aqueous ammonia (N, 18%) was used as soil fumigant, DCD (Dicyandiamide, Shanghai Aladdin Biochemical Technology Co., Ltd.) and NBPT (N-(N-butyl) thiophosphoric triamide, Shanghai Macklin Biochemical Technology Co., Ltd.) as nitrogen inhibitors. The experimental treatment included no soil fumigant (CK), aqueous ammonia (T1), aqueous ammonia + NBPT (T2), and aqueous ammonia + DCD (T3). The applied dose of aqueous ammonia was 750 L ha^−1^, while the doses of DCD and NBPT each accounted for 1% of aqueous ammonia dose. Commonly, three or five biological replicates are used in environmental microbiological studies to balance practicality and statistical power (Hugerth and Andersson, 2017). Each treatment contained three replicates. The soil microcosm bottles were closed with a rubber stopper equipped with a gas inlet and switch valve, and incubated at 28 °C.

### 2.3 Nitrous oxide collection and measurement

The gas samples were collected on the 1st, 2nd, 4th, 7th, 14th, 21st, and 28th days after sealing and fumigation to analyze the N_2_O emissions. The N_2_O emissions were measured using the modified methods by Harter et al. (2014) and Yan et al. (2015). At each scheduled sampling time, 5 mL headspace gas was withdrawn from each bottle using a gastight syringe (Agilent Technologies, USA) and transferred into 20 mL headspace vials pre-evacuated and purged with helium for subsequent N_2_O emission rate analysis. An Agilent 7697A gas chromatograph with an electron capture detector (63Ni-ECD) coupled with an Agilent 7694E headspace sampler was used to quantify the N_2_O concentrations. The gas chromatograph conditions and gas emission calculation method were described previously (Loftfield et al., 1997; Wang and Wang, 2003).

### 2.4 Soil sample collection and analysis

After gas sampling on the 1st, 7th, 14th, 21st, and 28th days after sealed fumigation, the stopper was removed and 15 ~ 20 g of soil was sampled using a sterile sampling spoon for physicochemical analysis and microbial ecological monitoring. Before being returned to the incubator, all the bottles were aerated using a vacuum pump and then resealed. If analysis was not conducted immediately, the soil samples were kept at 4 °C or −80 °C. For the extraction of root-knot nematode, the method described by the Baermann funnel method (Jenkins, 1964; Botelho et al., 2019) was employed. Soil sample was added to the tissue paper and placed on the funnel, filtered as water was added over the soil, incubated for 48 h, and the nematodes in the flat-bottomed glass test tube were collected. The number of root-knot nematode individuals in soil sample was determined under an inverted optical microscope.

### 2.5 Physical and chemical analysis

Soil ammonium nitrogen (NH_4_^+^-N) and nitrate nitrogen (NO_3_^−^-N) were extracted using a 2 M NaCl solution, and their concentrations were determined using a flow analyzer. Soil pH was measured using a pH meter (PHS-3C-02, Shanghai Sanxin Instrument Factory, China) with a soil-to-deionized water ratio of 1:2.5, whereas soil EC was measured using an EC meter (Spectrum Technologies Inc., USA) with a soil-to-deionized water ratio of 1:5. Soil moisture content was determined by drying the soil samples at 105 °C for 12 h to constant weight, and total nitrogen (TN) analysis was conducted using the Pu Biao Center Testing International Co., Ltd. (Tianjin, China).

### 2.6 Enzyme activity determination

Soil urease (UE), nitrate reductase (NR) and nitrite reductase (NiR) activities were measured using kits from Beijing Solarbio Science & Technology Co., Ltd., and all enzyme activities were quantified using a microplate reader (SPECTROStar NANO; Imgen Technologies, L.C., USA).

### 2.7 Nucleic acid extraction and quantitative amplification

The extraction of total soil DNA was performed with the FastDNA® Spin Kit for Soil (MP Biomedicals, USA), following the guidelines provided by the manufacturer. Using the SYBR Green I chimeric fluorescence method described by Wei et al. (2018), bacterial *16S rRNA* and fungal *ITS* genes were amplified and quantified. The primers and qPCR steps are outlined in Supplementary Table S1 (Orschler et al., 2019; Zhang et al., 2023). All qPCR experiments were conducted with a CFX96 Touch™ system (Bio-Rad Laboratories Inc., USA), with a reaction volume of 20 μL, consisting of 10 μL of 2 × ChamQ SYBR Color qPCR Master Mix (Vazyme Biotech Co., Ltd., China), 0.5 μL of each primer (10 μM), 1 μL of DNA template, and 8 μL of ddH_2_O. Serial 10-fold dilutions of plasmids harboring the target genes were used to generate standard curves, and all calibration curves demonstrated *R*^2^ values above 0.997. The gene copy number was standardized to the dry soil mass per gram, calculated using the following formula:


gene copy number=SQ×D×V/[S×(1−W)]

where SQ is the gene copy number measured by qPCR, D is the DNA dilution factor, V is the volume of extracted DNA (μL), S is the soil weight for DNA extraction (g), and W is the soil moisture content (Liu, 2018).

### 2.8 High-throughput sequencing and bioinformatics analysis

The V3–V4 region of the *16S rRNA* gene was amplified for bacterial analysis using the primer set 338F (5′-ACTCCTACGGGAGGCAGCAG-3′) and 806R (5′-GGACTACHVGGGTWTCTAAT-3′), while fungal communities were analyzed with the primers ITS-1F (5′-CTTGGTCATTTAGAGGAAGTAA-3′) and ITS-2R (5′-GCTGCGTTCTTCATCGATGC-3′). The PCRs were performed using the following protocol: denaturation at 95 °C for 3 min, followed by 27 cycles of 95 °C for 30s, 55 °C for 30 s, and 72 °C for 45 s, then a final extension at 72 °C for 10 min (PCR instrument: ABI GeneAmp® 9700). The PCR products were purified using 2% agarose gel and the AxyPrep DNA Gel Extraction Kit (Axygen Biosciences, Union City, CA, USA). Purified amplicons were sequenced in a paired end format using the Illumina MiSeq platform by Majorbio BioPharm Technology Co. Ltd. (Shanghai, China). First, the raw reads were trimmed by using Fastp to obtain the clean data. Then the obtain reads were assembled into contigs individually for each sample through software Megahit. Then, the software MetaGene was employed to open reading frames (ORFs) annotation (Noguchi et al., 2006). The annotation of obtained genes was performed using software BLASTP for functional and taxonomic analyses (Kanehisa and Goto, 2000). The microbial nitrogen cycle-related genes were identified using KO numbers, and the abundance of N cycle-related genes was quantified using TPM values. Sequencing raw sequences were uploaded to NCBI Website as BioProject SRP566419.

### 2.9 Data analysis

Data analyses were performed using the SPSS statistical software (Version 25.0). Differences in soil parameters, nitrous oxide emission, nematode population, enzymatic activity, gene abundances and microbial characteristics among the treatments were assessed by One-way Analysis of Variance (ANOVA). Significant differences between treatments were assessed using Tukey’s HSD test. Graphics were generated using Origin (version 2024b). After quality control, 5,125,148 high-quality bacterial 16S rRNA V3-V4 sequences and 4,706,180 high-quality fungal ITS sequences were obtained. OTU clustering was performed at a 97% similarity for non-repeating sequences (excluding singletons). The OTU clustering of all sequences was performed using Uparse (version 7.0), while the alpha diversity analysis of soil microorganisms on day 28 was conducted using Mothur (version 1.30.2). Based on the taxonomic analysis, the species composition of different groups at the phylum and genus levels was visualized using R (version 3.3.1). Principal coordinate analysis (PCoA) was performed and visualized in R to assess similarities and differences in the sample community composition. Employing community abundance data, the Kruskal-Wallis rank sum test (Kruskal–Wallis H test) was utilized to detect species exhibiting significant variations in abundance across the samples. Redundancy analysis (RDA) and corresponding visualizations were conducted using the vegan package in R (version 2.4.3) to illustrate the relationships between sample distribution and environmental factors. Additionally, Spearman correlations between dataset variables were evaluated and visualized using Origin software.

## 3 Results

### 3.1 Effects of ammonia on soil pH, EC and root-knot nematodes

In this study, aqueous ammonia and inhibitor treatments significantly altered the soil properties, increasing the soil pH and EC compared with CK. The pH and EC values in T2 and T3, which included inhibitors, were notably higher than those in T1 (Supplementary Table S2). Nematode populations were reduced under all treatments by the 28th day post-fumigation. Treatments with T1, T2, and T3 exhibited significant declines relative to the control (CK), with control efficacy exceeding 64% in each treatment. T1 exhibited the highest efficacy, surpassing those of T2 and T3 by 11.68% and 8.31%, respectively.

### 3.2 Nitrogen dynamics and conservation

**Figure 1:**
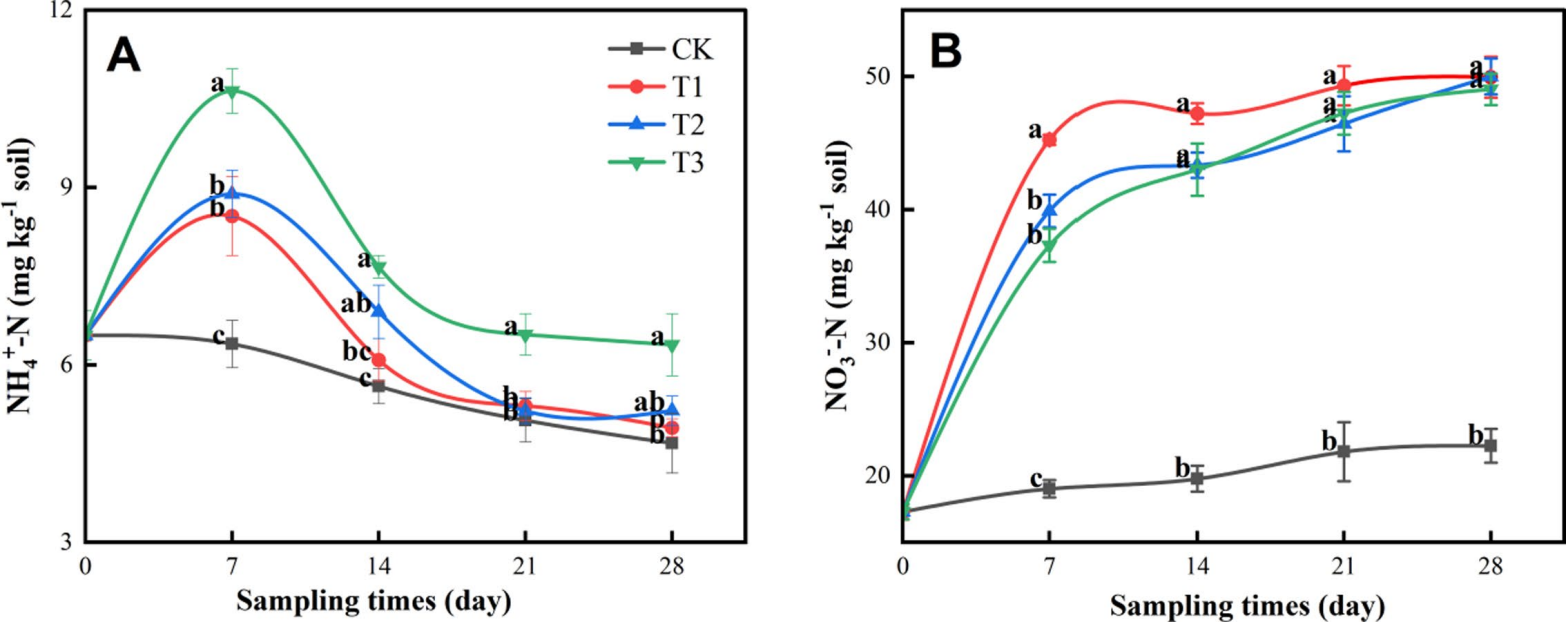
Dynamics of soil NH_4_^+^-N **(A)** and NO_3_^−^-N **(B)** concentrations. Significant differences (Tukey’s HSD, *p* < 0.05) among the different treatments are shown by different letters.

During the 4 weeks of fumigation, T3 maintained the highest NH_4_^+^-N concentration, peaking at 10.63 mg kg^−1^ in the first week as the increase from 6.5 mg kg^−1^ on day 0, exceeding T1, T2, and CK (Figure 1A). Even after 28 d, T3 continued to exhibit the highest NH₄^+^-N concentration, whereas the NO_3_^−^-N concentrations followed an inverse pattern (Figure 1B). After the 7th day, the NH_4_^+^-N concentrations rapidly declined across all treatments, whereas the NO_3_^−^-N concentrations gradually increased. Within the first 7 days, the application of nitrification inhibitor markedly decreased nitrate nitrogen (NO₃^−^-N) concentrations relative to the aqueous ammonia-only treatment. The NO^3−^-N concentration in T1 increased from 17.29 to 45.24 mg kg^−1^ from day 0 to day 7, while treatments T2 and T3 exhibited weaker increases and were 11.8% and 17.55% lower than T1, respectively. Both NBPT and DCD inhibited the soil conversion of NH_4_^+^-N to NO_3_^−^-N following ammonia application, with DCD showing slightly stronger inhibition.

### 3.3 Soil N_2_O emissions

**Figure 2:**
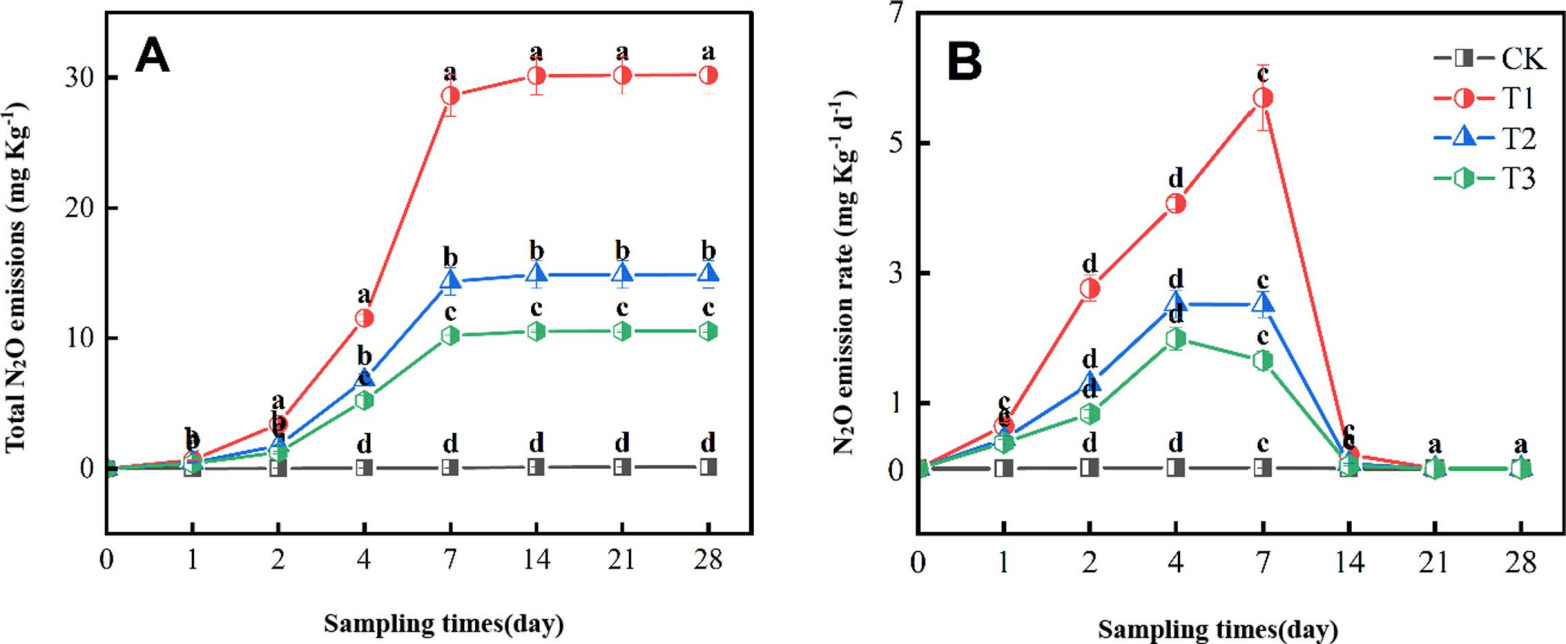
Cumulative N_2_O emissions **(A)** and its emission rate in particular days **(B)**. Significant differences (Tukey’s HSD, *p* < 0.05) among the different treatments are shown by different letters.

Figure 2 illustrates the N_2_O production rates and total cumulative emissions observed during the laboratory incubation. Aqueous ammonia fumigation increased overall N_2_O production, whereas the suppression treatments effectively reduced N_2_O emissions. The total N_2_O emissions in T1 were notably greater compared to those in CK, T2 and T3 (Figure 2A). After 1 week, the N_2_O emission rate in all treatment groups declined, returning to the control levels by approximately the 21st day (Figure 2B). During this decline, the influence of ammonia remained significant, with the N_2_O release rate in the soil treated solely with ammonia (T1) still exceeding that of the other treatments (*p* < 0.05). Among the treatments with inhibitors, T3 exhibited the optimal control efficacy against N_2_O emissions, with an inhibition rate 65.09%, which was 14.4% higher than that of T2.

### 3.4 Soil enzyme activity

**Figure 3:**
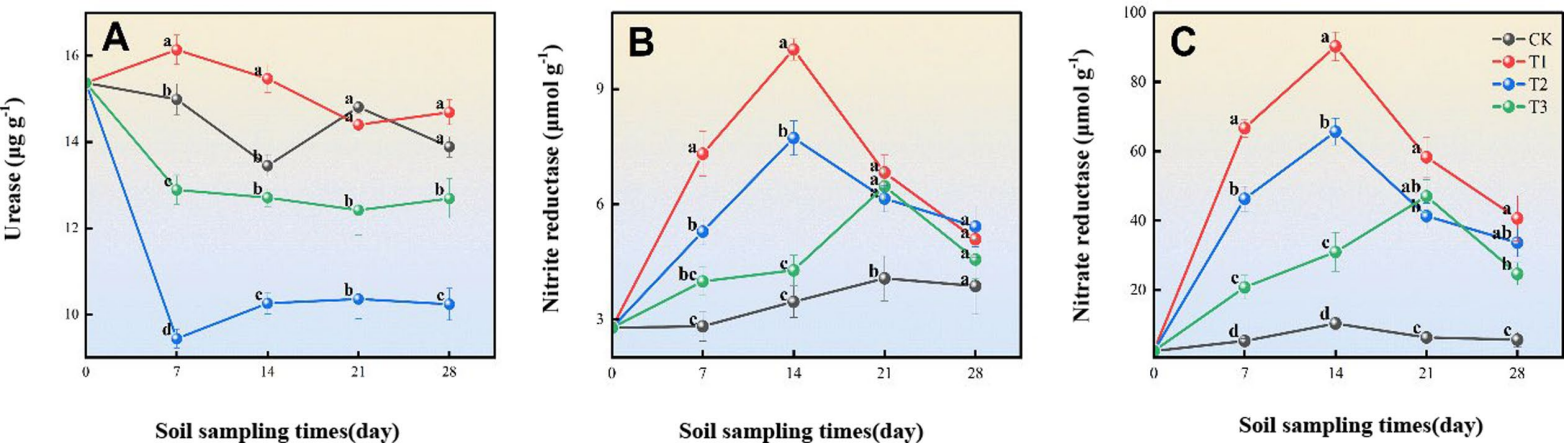
Dynamic changes in urease **(A)**, nitrite reductase **(B)**, and nitrate reductase **(C)** during fumigation. Significant differences (Tukey’s HSD, *p* < 0.05) among the different treatments are shown by different letters.

Compared with CK, aqueous ammonia application increased the activities of UE, NiR, and NR. Both inhibitors exhibited varying effects on mitigating the influence of aqueous ammonia on enzyme activity. Throughout the fumigation period, the addition of NBPT in T2 significantly reduced UE activity, with a reduction range of 13.64% to 20.17% relative to T1 (Figure 3A), whereas DCD in the T3 treatment significantly decreased NiR activity by 5.34%–57.40% (Figure 3B). Similarly, NR activity in T3 was 19.37% to 68.86% lower than that in T1 (Figure 3C). The effects of T1, T2, and T3 on NiR and NR activities were generally similar. Before the 14th day, the NiR and NR activities in T3-treated soil were significantly lower than those in T1 and T2, with T3 delaying the activation of both enzymes. By the 21st day, no significant differences in enzyme activity were observed among T1, T2, and T3.

### 3.5 Quantification of bacteria, fungi populations, and abundance of genes encoding key enzymes involved in nitrogen cycling

**Figure 4:**
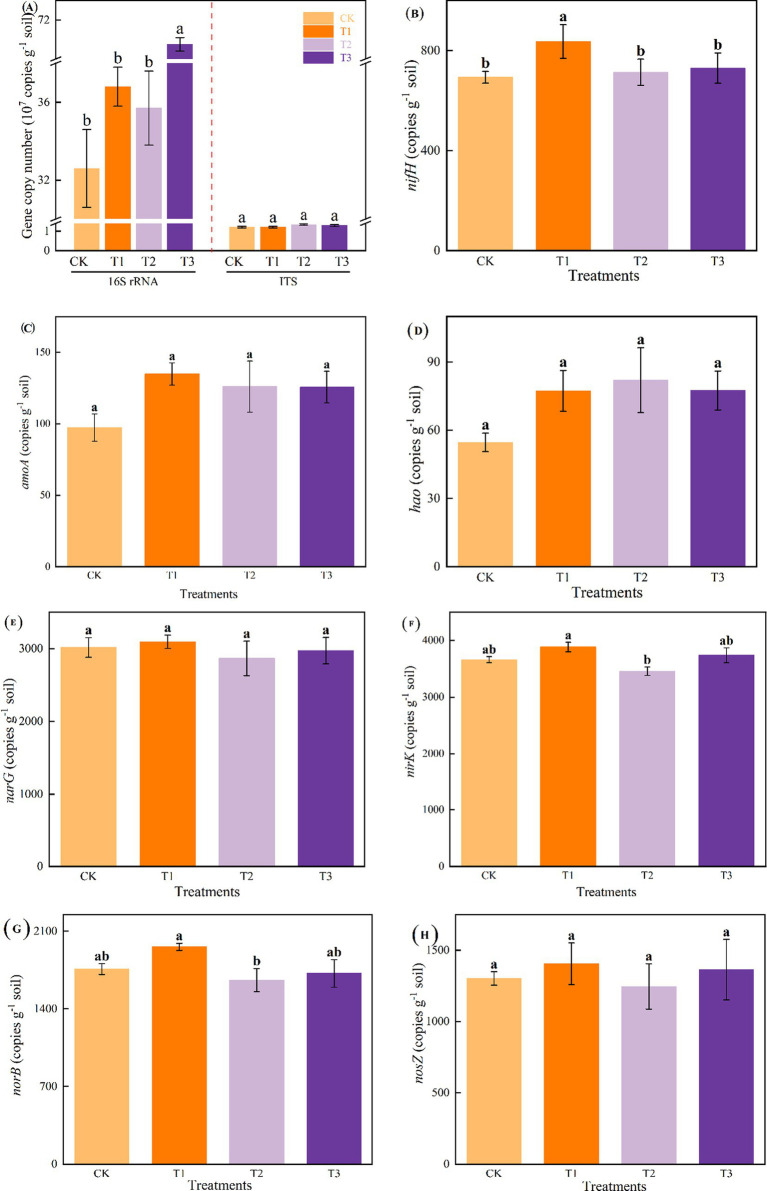
Absolute abundance of total bacteria (**A**; *16S rRNA*), total fungi (**A**; *ITS*), and nitrogen cycle-related genes, including nitrogen fixation (**B**; *nifH*), nitrification (**C,D**; *amoA* and *hao*), and denitrification (**E–H**; *narG*, *nirK*, *norB*, and *nosZ*). Significant differences (Tukey’s HSD, *p* < 0.05) among the different treatments are shown by different letters. *nifH*, nitrogenase gene; *amoA*, ammonia monooxygenase gene; *hao*, hydroxylamine oxidoreductase gene; *narG*, nitrate reductase gene; *nirK*, nitrite reductase gene; *norB*, nitric oxide reductase gene; *nosZ*, nitrous oxide reductase gene.

Figure 4 illustrates the total abundance of bacteria and fungi, together with the relative abundances of key functional genes governing nitrogen cycling, including the nitrogen-fixing gene *nifH*, nitrification genes (*amoA*, *hao*), and denitrification genes (*narG*, *nirK*, *norB*, *nosZ*). After fumigation, no significant differences were observed in fungal *ITS* gene copy numbers among treatments (*p* > 0.05). However, the *16S rRNA* gene copy number in the T3 soil was significantly higher than that in the other treatments (*p* < 0.05) (Figure 4A). T1 had the highest *nifH* gene abundance, significantly exceeding those of CK, T2, and T3 (*p* < 0.05) (Figure 4B). Although no significant differences were identified in the abundance of *amoA* and *hao* genes related to nitrification, their levels in T1, T2, and T3 were higher than those in CK (Figures 4C,D). Regarding denitrification, the abundance of *nirK* and *norB* genes was significantly different between the T2 and T1 soils (*p* < 0.05). Compared with CK, T3 had no significant effect on *nirK* and *norB* expression (Figures 4F,G), and no significant differences were observed in the abundance of *narG* and *nosZ* genes across all treatments (Figures 4E,H).

### 3.6 Microbial diversity and community structure

**Figure 5:**
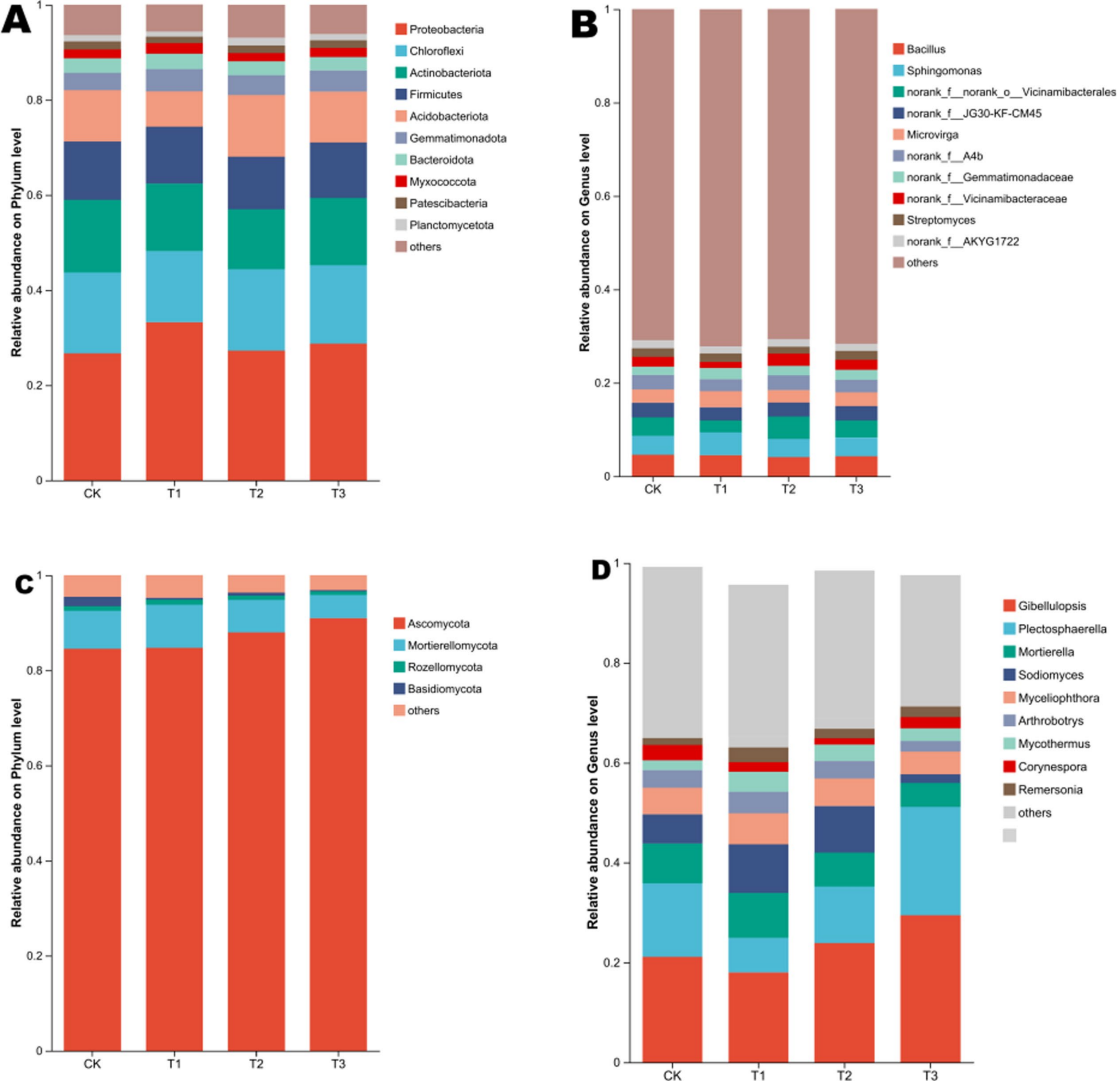
The composition of the top 10 species of microorganisms within the treatments. Bacterial composition at the phylum level **(A)**, Bacterial composition at the genus level **(B)**, Fungal composition at the phylum level **(C)**, and Fungal composition at the genus **(D)**.

Soil bacterial community analysis identified 35 phyla and 683 genera, with the dominant phyla across all treatments being *Proteobacteria*, *Chloroflexi*, *Actinobacteriota*, *Firmicutes*, and *Acidobacteriota*, which collectively accounted for > 80% of the total bacterial community (Figure 5A). Among the top 20 bacterial genera, those involved in nitrogen (N) transformation included *Bacillus*, *Sphingomonas*, *Gemmatimonas*, and *Streptomyces*, belonging to *Firmicutes*, *Pseudomonas*, *Gemmonada*, and *Actinobacteria*, respectively (Figure 5B). In the T1 treatment, the relative abundance of *Proteobacteria* was higher than in other treatments, accounting for more than 33% of the total bacterial community. Additionally, the relative abundance of *Sphingomonas* was higher in T1 than in other treatments. Nevertheless, there were no significant differences among all treatments (*p* > 0.05).

The fungal community was comprised of 13 phyla and 309 genera, with *Ascomycota* and *Mortierellomycota* dominating at the phylum level, representing over 90% of the fungal species (Figure 5C). At the genus level, *Gibellulopsis*, *Plectosphaerella*, and *Mortierella* were the most abundant genera (Figure 5D). Treatments T1, T2, and T3 reduced the relative abundance of *Basidiomycota* within fungal communities. Compared with the CK treatment, the relative abundances of *Gibellulopsis*, *Plectosphaerella* and *Corynespora* decreased in T1, whereas the relative abundances of *Sodiomyces* increased. In T3, ammonia combined with DCD significantly increased the relative abundance of *Gibellulopsis* and *Plectosphaerella*, whereas that of *Mortierella* and *Sodiomyces* decreased.

**Figure 6:**
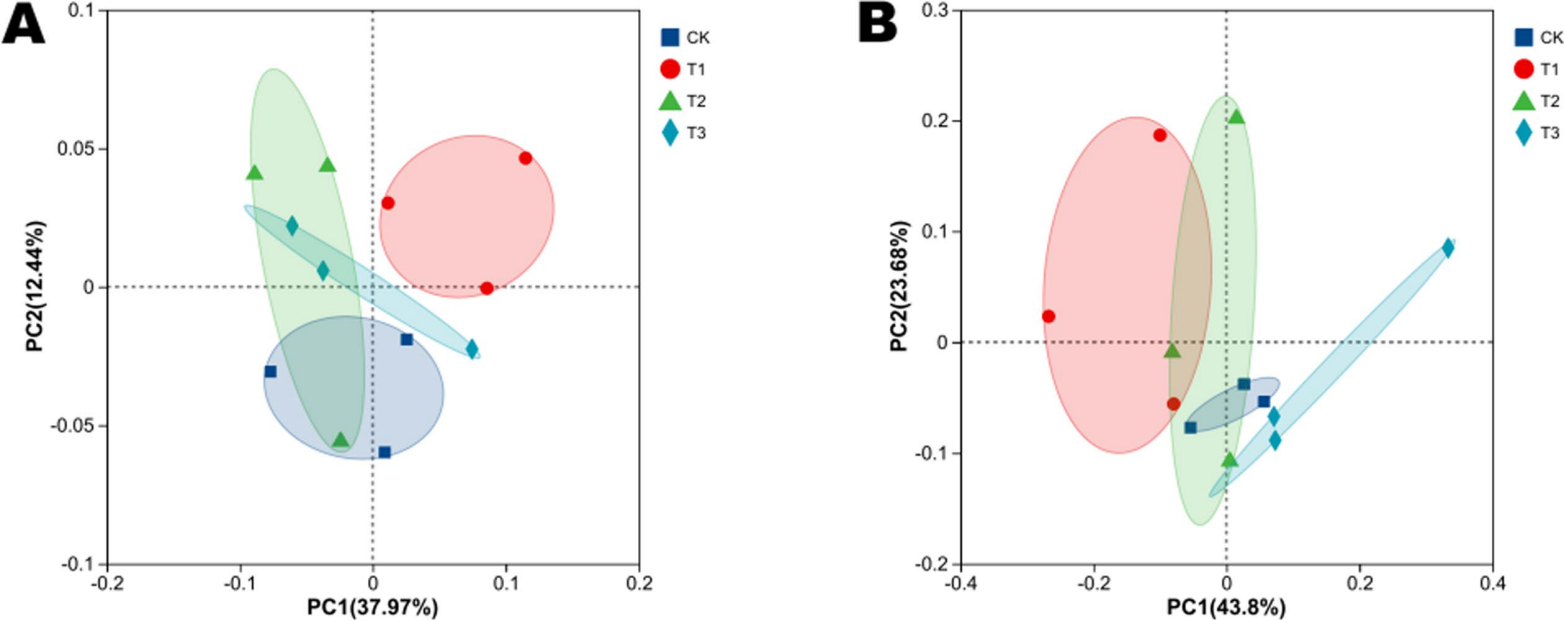
Principal coordinate analysis (PCoA) of bacterial **(A)** and fungal **(B)** microbial community composition at the genus level.

The Shannon and Coverage indices indicated no significant differences in bacterial and fungal alpha diversity among treatments with ammonia and inhibitors (Supplementary Figures S1B,C,E,F). However, the Sobs index showed that the fungal richness in T1, T2, and T3 was significantly or very significantly lower than that in CK (Supplementary Figure S1D), whereas the bacterial richness remained unaffected (Supplementary Figure S1A). PCoA at the genus level revealed that the microbial community structure in the T1 soil differed from that of other treatments, whereas the structures in T2 and T3 were similar to those in the control (Figure 6).

In the bacterial community, *Alphaproteobacteria* exhibited a significantly higher relative abundance in the T1 treatment than in CK, T2, and T3, whereas norank_f_*Gemmataceae* was more abundant in T2 than in T1 (Supplementary Figures S2A). Additionally, norank_f_*Longimicrobiaceae* exhibited the highest relative abundance in T1 compared with the other treatments. In the fungal community, *Plectosphaerella* was significantly more abundant in T3 than in the other treatments, whereas *Keratinophyton* displayed the highest relative abundance in T1 (Supplementary Figures S2B).

### 3.7 Correlation analysis of soil environmental factors

**Figure 7:**
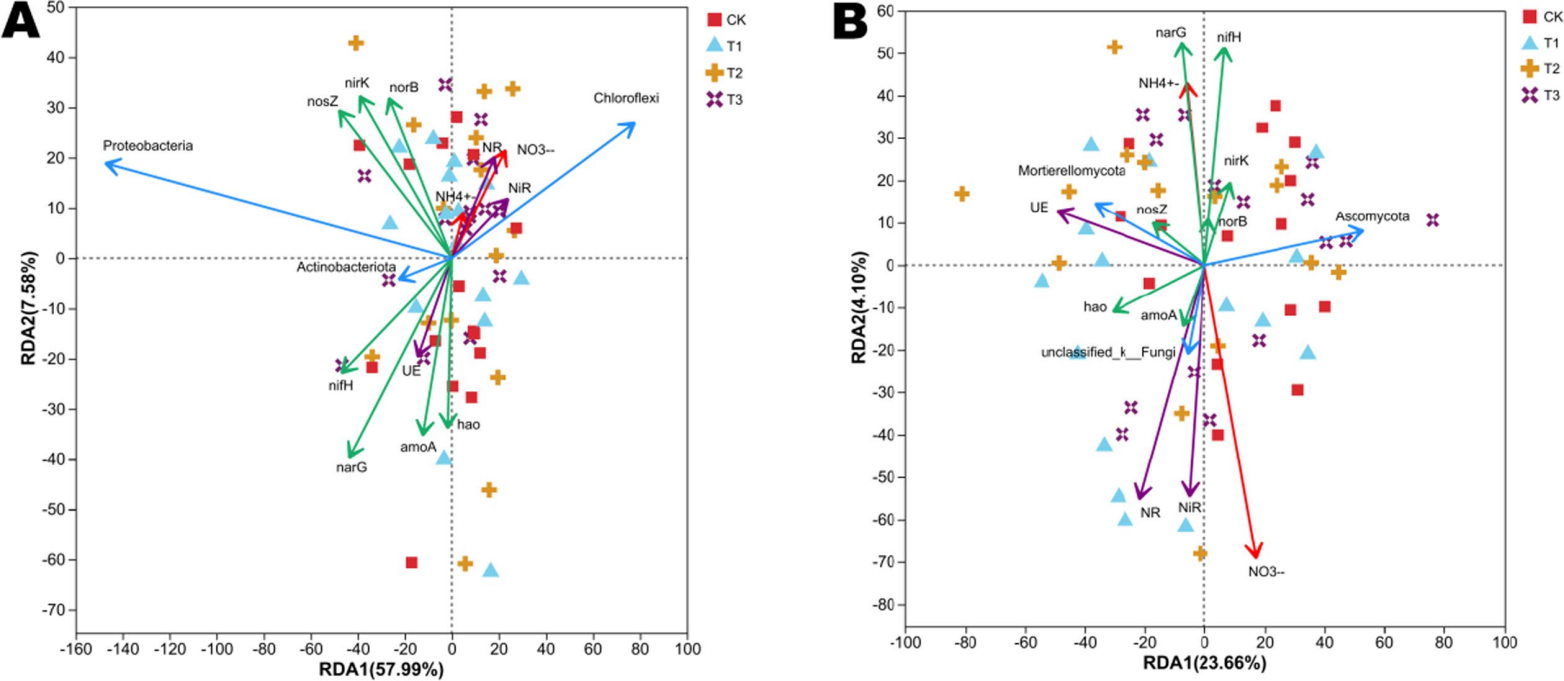
The relationship between the top 3 bacterial **(A)** and fungal **(B)** species at the phylum level and soil physicochemical properties as well as functional genes. Red arrows-soil properties, blue arrows -dominant species, green arrows-nitrogen cycle functional genes, and purple arrows-soil nitrogen cycle-related enzyme activities.

**Figure 8:**
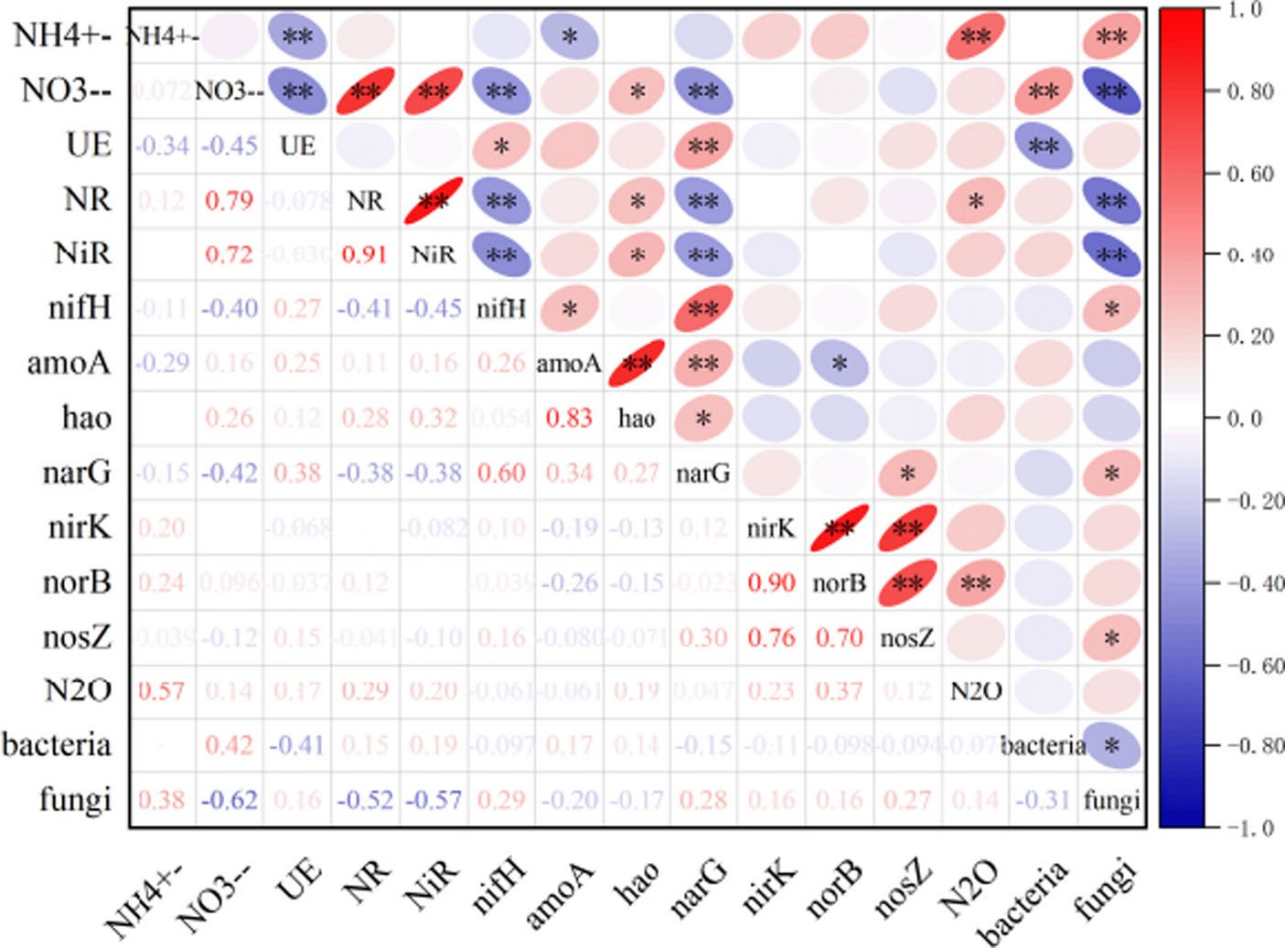
The matrix of the correlation between nitrous oxide and environmental factors, enzyme activities, and functional genes. Red represents positive correlations, while blue represents negative correlations. Asterisks in the color blocks represent significance levels, * denotes 0.01 < *p* ≤ 0.05; ** denotes 0.001 < *p* ≤ 0.01.

RDA was conducted to assess the effects of soil properties on microbial communities, nitrogen cycle-related genes, and soil enzyme activities. Soil characteristics accounted for 65.57 and 27.76% of the variation in the bacterial and fungal community structures, respectively (Figures 7A,B). Among these factors, NO_3_^−^-N was identified as a key driver influencing NR, NiR, and Chloroflexi abundance in bacterial communities. Both NO_3_^−^-N and NH_4_^+^-N were positively correlated with the denitrification genes *nosZ*, *nirK*, and *norB*, whereas the nitrogen fixation (*nifH*) and nitrification (*amoA*, *hao*) genes exhibited strong negative correlations with these nitrogen compounds (Figure 7A). In the fungal communities, the unclassified fungal species, NR, NiR, and the nitrification genes *hao* and *amoA* were positively correlated with soil NO_3_^−^-N concentrations, and their variation was mainly influenced by NO_3_^−^-N levels. In contrast, nitrogen fixation (*nifH*) and denitrification genes (*narG*, *nirK*, *norB*, and *nosZ*) were negatively correlated with NO_3_^−^-N (Figure 7B). Figure 8 illustrates the correlations among soil microbial abundance, physicochemical properties, nitrogen cycle genes, enzyme activities, and N_2_O emission rates. N_2_O emission rates exhibited a highly significant positive correlation with NH_4_^+^-N concentration (*r* > 0.57, *p* < 0.01) and a significant positive correlation with NR activity and *norB* abundance (*r* > 0.29, *p* < 0.05; *r* > 0.37, *p* < 0.01). The abundance of bacterial and fungal microorganisms showed highly significant positive correlations with NO_3_^−^-N and NH_4_^+^-N concentrations, respectively (*r* > 0.42, *p* < 0.01; *r* > 0.38, *p* < 0.01). The nitrogen fixation gene *nifH* demonstrated a highly significant negative correlation with NO_3_^−^-N (*r* < −0.40, *p* < 0.01), whereas the nitrification gene *amoA* was significantly negatively correlated with NH_4_^+^-N (*r* < −0.29, *p* < 0.01).

## 4 Discussion

This study demonstrated that both ammonia alone and in combination with inhibitors effectively controlled root-knot nematodes, with T1 treatment (aqueous ammonia fumigation alone) achieving a maximum control rate of 75.73%. Previous studies reported that nitrogen substances can control root-knot nematodes. This may be attributed to the changes in soil pH and salinity induced by nitrogen fertilizers, which affect nematode populations; additionally, nitrogen components exert direct lethal effects on nematodes (Shakeel et al., 2022). The present study also revealed that nitrogen application increased soil ammonium nitrogen content, which in turn raised soil pH and EC values. These changes are likely correlated with nematode suppression (Figure 1; Supplementary Table S1). Further speculation suggests that exposure to nitrogen environments may damage the intestinal structure of root-knot nematodes. Meanwhile, nitrogen fertilizers can accelerate the metabolism of second-stage juveniles (J2), resulting in faster depletion of their lipid reserves compared with the untreated control group. This reduces the energy required for nematodes to locate and infect host plants, ultimately leading to their death (Shakeel et al., 2020).

N_2_O is a major ozone-depleting substance, and agricultural soils are a primary source of anthropogenic emissions (Reay et al., 2012). Previous studies have shown that soil fumigants can influence N_2_O production to varying extents. For instance, CP fumigation suppresses the expression of genes involved in N_2_O production and consumption while shifting the dominant N_2_O generation pathway from nitrification to denitrification, resulting in emissions 23 to 25 times higher than that of the control (Fang et al., 2018a,b). Similarly, dazomet (DZ) fumigation can significantly stimulate potential denitrification activity, leading to increased N_2_O emissions (Yan et al., 2015). In contrast, fumigation with 1,3-D and DMDS has no significant effect on N_2_O production (Yan et al., 2015). In this study, the application of inhibitors effectively reduced N_2_O emission, thereby mitigating the environmental effects of ammonia disinfection.

Ammonia water treatment significantly affected soil properties by increasing pH and EC, resulting in an alkaline environment. Additionally, the combined application of ammonia and inhibitors effectively regulated soil NH_4_^+^-N and NO_3_^−^-N concentrations, slowed the conversion of NH_4_^+^-N into NO_3_^−^-N, and inhibited the nitrification process. This trend aligns with previous research findings (Frame, 2017). Extensive research has indicated that nitrification inhibitors can significantly reduce the nitrification rate of nitrogen in soil compared with untreated nitrogen fertilizers. Studies with similar trial durations have consistently demonstrated that these inhibitors effectively delay the conversion of nitrogen fertilizers by suppressing the activity of key microorganisms involved in the nitrification process (Soares et al., 2012; Zaman et al., 2008).

Soil microbes are essential indicators of soil health (Thiele-Bruhn et al., 2020). This experiment revealed that fumigation did not significantly affect fungal *ITS* gene copy numbers across treatments (*p* > 0.05). However, the *16S rRNA* gene copy number in soils treated with DCD (T3) was significantly higher than that of the other treatments (*p* < 0.05). Previous studies have shown that nitrification inhibitors can promote the growth of specific bacterial groups such as *Nitrosomonas* by regulating the availability of soil nitrogen sources (Zhang and Agr, 2018). Although the microbial diversity in T3 did not differ significantly from that in the other treatments, the notable increase in bacterial abundance aligned with previous findings. This effect may be due to the influence of DCD on the conversion of ammonia into nitrogen forms that certain soil bacteria can utilize, thereby promoting their growth and proliferation (Li et al., 2023).

In the soil of each treatment group, *Proteobacteria* was the dominant bacterial phylum, followed by *Chloroflexi*, while *Bacillus* was the dominant bacterial genus. The relative abundance of *Proteobacteria* in the T1 treatment was higher than that in the other treatments, whereas *Acidobacteriota* and *Vicinamibacterales* were less abundant. *Proteobacteria* play a crucial role in organic biodegradation and nitrogen (N) transformation, with most nitrifying and denitrifying microorganisms belonging to the phyla *Proteobacteria*, *Firmicutes*, and *Actinobacteria* (Guo et al., 2020). *Acidobacteriota* are typically oligotrophic and acidophilic bacteria that exhibit a negative correlation with soil pH (Weng et al., 2023). Previous studies have shown that nitrogen fertilizer application can reduce the relative abundance of *Acidobacteriota* (Dai et al., 2018), which is consistent with observations in the T1 treatment. Additionally, nitrogen inputs have been found to inhibit the growth of oligotrophic bacteria, including certain *Acidobacteria* (Leff et al., 2015). This process exhibits the potential to decompose nutrient-poor and recalcitrant substrates. However, it also promoted the growth of copiotrophic bacteria, such as certain *Alproteobacteria* and *Actinobacteria*, which thrived owing to their rapid growth rates and lower C use efficiency (Ling et al., 2017; Zhou et al., 2015). Within the fungal community, the relative abundances of *Gibellulopsis*, *Plectosphaerella* and *Corynespora* were significantly lower under T1 treatment than in CK. Consistent with previous reports, these genera are known phytopathogens (Carlucci et al., 2012; Raimondo and Carlucci, 2018; Burhanah et al., 2019; Han et al., 2020; Yulduzkhon et al., 2024). Our results confirmed that ammonia fumigation exerts selective inhibitory effects and effectively suppresses the above phytopathogenic fungi rather than all fungal taxa in soil. However, the addition of DCD to ammonia fumigated soil decreased the efficacy of the ammonia fumigation in reducing plant pathogens, as indicated by the greater fold increase in the relative abundances of *Gibellulopsis*, *Plectosphaerella* and *Corynespora*. In general, DCD suppresses ammonium nitrification, mitigating volatilization and slowing the transformation from NH_4_^+^-N to NO_3_^−^-N (Tian et al., 1998). Accordingly, DCD amendment facilitates obvious NH_4_^+^-N accumulation and reduces soil NO_3_^−^ content. Previous research reported that DCD could weaken the antifungal efficacy of ammonium bicarbonate against *Fusarium oxysporum*, and the pathogen-inhibiting capacity of ammonium largely relies on its metabolite nitrous acid (Sun et al., 2015). In our experiment, T3 achieved significantly higher NH_4_^+^-N but lower NO_3_^−^ relative to other treatments, and these variations align with the pathogen-suppressing properties of ammonium-based fumigation.

Soil properties are closely associated with functional nitrogen-cycling genes and microbial community structure. These factors collectively affect the rate of N_2_O production. RDA and Spearman correlation analyses revealed that the bacterial and fungal community structures shown significant positive correlated with soil NO_3_^−^-N and NH_4_^+^-N concentrations, respectively. This finding aligns with those of Sun et al. (2015, 2020) and Yuan et al. (2021) who demonstrated that soil NH_4_^+^-N concentration significantly influenced the microbial community structure. Furthermore, Fang et al. (2018a,b) suggested that fumigation suppressed the functional gene *amoA*, contributing to increased NH_4_^+^-N and decreased NO_3_^−^-N concentrations. In this study, the accumulation of NH_4_^+^-N in the treatment groups (T1, T2, and T3) may be attributed to the lack of significant increases in *amoA* and *hao* gene expression, which reduced ammonia oxidation (Figure 4C). Moreover, in the T3 treatment, the NH_4_^+^-N concentration and bacterial *16S rRNA* gene copy number were significantly higher than those in the other treatments, contradicting previous findings and warranting further investigation. As shown in Figure 8, the abundance of the *norB* gene was positively correlated with NH_4_^+^-N concentration and N_2_O emission rates, implying that *norB* serves as a key denitrifying functional gene regulating soil nitrogen dynamics via metabolic feedback (Yang et al., 2024). The tight linkage between N_2_O production and potential denitrification suggests intermediate substances from fumigant degradation supply nitrogen substrates for microbial denitrification (Yan et al., 2015), which stimulates denitrifier activity and accelerates soil N_2_O generation (Rojas-Oropeza et al., 2012).

Several inherent limitations of this laboratory microcosm study should be acknowledged. First, all experiments were performed in sealed glass bottles, which created constrained aeration and altered gas diffusion conditions compared with open field soil environments. Such closed incubation systems may induce artificial microbial responses and deviate from in-situ soil biochemical processes. Second, this study was conducted under controlled laboratory conditions without field-scale validation, and the actual field effects of ammonia fumigation may be affected by complex environmental factors. Third, the short-term 28-day incubation could not fully reflect the long-term resilience and stability of soil microbial communities and N_2_O emission characteristics after fumigation. Additionally, agricultural factors including crop growth, field fertilization, and irrigation management were excluded from our microcosm system, which play critical roles in regulating rhizosphere microbial flora and soil nitrogen cycling. Therefore, further pot, greenhouse, and field trials are required to verify the practical effects and underlying mechanisms of ammonia fumigation on soil microbial regulation and nitrogen dynamics under actual agricultural conditions.

## 5 Conclusion

Our microcosm experiment indicated that aqueous ammonia fumigation could effectively suppress RKNs and mitigate N_2_O greenhouse gas emissions. Compared with the initial nematode population density, the nematode density was reduced by 75.73%, which was the highest reduction rate across all treatments. When combined with inhibitors such as DCD, it further mitigated the N_2_O emissions by regulating soil NO_3_^−^-N and NH_4_^+^-N concentrations. Additionally, Aqueous ammonia fumigation increased soil pH and electrical conductivity, enhanced nitrate and nitrite reductase activities, and significantly suppressed pathogenic fungi (e.g., *Plectosphaerella*). Quantitative PCR quantification of 16S rRNA and ITS gene copies revealed no significant differences in total absolute microbial abundance between ammonia-fumigated soil and the non-fumigated control (*p* > 0.05). Fumigation significantly reduced fungal richness and reshaped overall microbial community composition, yet bacterial alpha diversity remained unaltered across treatments. In addition, the relative abundances of functional genes involved in nitrogen cycling differed markedly among treatments. These findings demonstrate that total microbial biomass remained stable, even amid pronounced restructuring of microbial assemblages. The present findings are based on laboratory microcosm experiments. Therefore, the potential of aqueous ammonia as an environment-friendly soil disinfection strategy needs further verification under field conditions. Future studies are required to clarify the persistence of ammonia effects, as well as its comprehensive influences on soil microbial diversity, community structure, ecological functions, and crop performance under practical agricultural scenarios.

## Data Availability

The original sequencing datasets generated in this study are publicly available. The data can be accessed at: https://www.ncbi.nlm.nih.gov/sra/PRJNA1228553.
