# Supplementary material for: Ammonia fumigation combined with nitrogen inhibitor reduces root-knot nematode populations and N2O emission but not bacterial alpha diversity
Source: Front Microbiol. 2026 Jul 22;17:1756683. doi: 10.3389/fmicb.2026.1756683 (PMC13437642; doi:10.3389/fmicb.2026.1756683)
Supplement: Supplementary file 1 [file Table_1.DOCX]

| Specificity | Primer | Sequence (5’-3’) | Thermal profiles | References |
| --- | --- | --- | --- | --- |
| 16S rRNA | 338F | ACTCCTACGGGAGGCAGCAG | 30 s at 95°C, 40 cycles consisting of 10 s at 95°C, 30 s at 60°C and melt curve 65°C to 95°C, increment 0.5°C. | Orschler *et al*., 2019 |
|  | 518R | ATTACCGCGGCTGCTGG |  |  |
| ITS rRNA | ITS-1F | CTTGGTCATTTAGAGGAAGTAA | 30 s at 95°C, 40 cycles consisting of 5 s at 95°C, 30 s at 58°C and melt curve 65°C to 95°C, increment 0.5°C. | Zhang *et al*., 2023 |
|  | ITS-2R | GCTGCGTTCTTCATCGATGC |  |  |

Table S1 Primers used for quantitative amplification in this study and their corresponding reaction profiles.

| ID | RNK (Individuals / 100g dry soil) | EC (mS cm^-1^) | pH |
| --- | --- | --- | --- |
| CK | 670.67 ± 59.15 a | 0.43 ± 0.01 c | 7.0 ± 0.01 d |
| T1 | 221.00 ± 60.00 b | 0.53 ± 0.01 b | 7.6 ± 0.01 c |
| T2 | 327.33 ± 27.49 b | 0.56 ± 0.01 a | 7.7 ± 0.01 b |
| T3 | 296.67 ± 44.43 b | 0.55 ± 0.01 a | 7.8 ± 0.01 a |

Table S2 Number of root-knot nematodes, pH and EC values in soil after fumigation. The different lowercase letters at the end of each column represent a significant difference between treatments according to Tukey’s HSD test (*p* < 0.05).

|  |  |  |
| --- | --- | --- |
|  |  |  |

Figure S1 Soil microbial biodiversity indices in 28th day after fumigation (A) Bacterial Sobs index, (B) Bacterial Shannon index, (C) Bacterial Coverage index, (D) Fungal Sobs index, (E) Fungal Shannon index, and (F) Fungal Coverage index. * denotes 0.01 <*p* ≤ 0.05, ** denotes 0.001 < *p* ≤ 0.01.

Figure S2 The most abundant top 10 genus of (A) Bacteria; (B) Fungi. * denotes 0.01 < P ≤ 0.05.
